# Biofuel Potential of Plants Transformed Genetically with *NAC* Family Genes

**DOI:** 10.3389/fpls.2016.00022

**Published:** 2016-01-26

**Authors:** Sadhana Singh, Atul Grover, M. Nasim

**Affiliations:** Biotechnology Division, Defence Institute of Bio-Energy ResearchHaldwani, India

**Keywords:** *NAC*, genetically engineered plants, abiotic stress tolerance, secondary growth, cell wall synthesis, biomass

## Abstract

*NAC* genes contribute to enhance survivability of plants under conditions of environmental stress and in secondary growth of the plants, thereby building biomass. Thus, genetic transformation of plants using *NAC* genes provides a possibility to tailor biofuel plants. Over-expression studies have indicated that *NAC* family genes can provide tolerance to various biotic and abiotic stresses, either by physiological or biochemical changes at the cellular level, or by affecting visible morphological and anatomical changes, for example, by development of lateral roots in a number of plants. Over-expression of these genes also work as triggers for development of secondary cell walls. In our laboratory, we have observed a *NAC* gene from *Lepidium latifolium* contributing to both enhanced biomass as well as cold stress tolerance of model plants tobacco. Thus, we have reviewed all the developments of genetic engineering using *NAC* genes which could enhance the traits required for biofuel plants, either by enhancing the stress tolerance or by enhancing the biomass of the plants.

## Introduction

**FIGURE 1:**
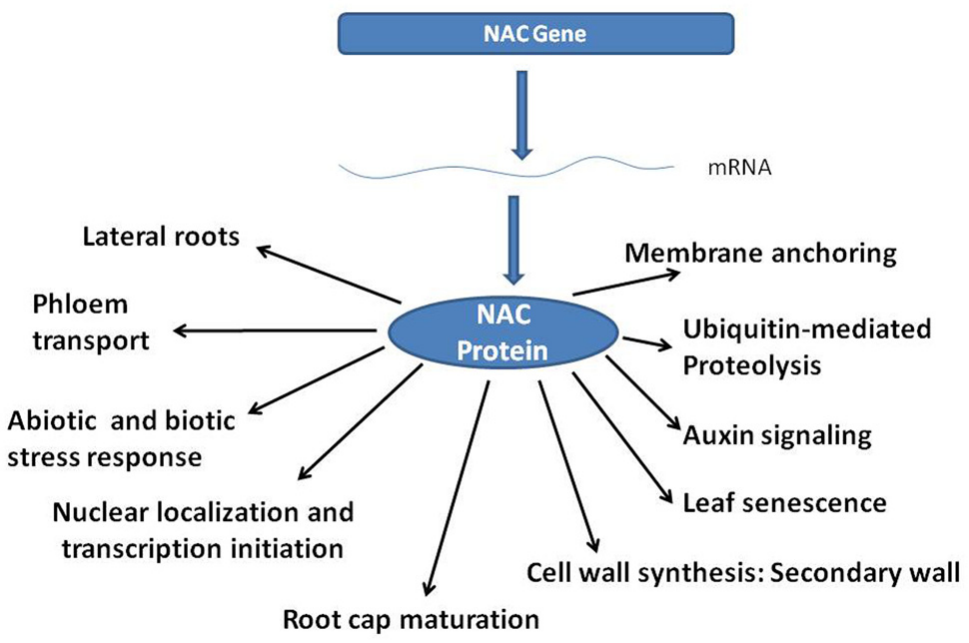
**Diversity of functions into which NAC genes and proteins are involved in**.

*NAC* (*NAM, ATAF, CUC*) genes containing the NAC domain, constitute one of the largest plant-specific transcription factor (TF) families. NAC family TFs are characterized by a highly conserved N-terminal DNA binding domain (Olsen et al., 2005) and a diversified C-terminal domain that generally regulates the transcriptional activation (Yamaguchi et al., 2008). Functional characterization of the *NAC* family genes using over-expression studies (He et al., 2005) have helped understanding various biological roles of NAC proteins as indicated in **Figure 1** Interestingly, *NAC* genes contribute to enhanced survivability of plants under stress (Puranik et al., 2012), and in secondary growth of the plants (Nakano et al., 2015), thereby building biomass.

Biofuel plants of the future, so-called fourth generation biofuels are perceived to be the ones that survive under harshest of conditions owing to their abilities to withstand stress, and produce large amounts of biomass that can be converted to green diesel, via biomass-to-liquid technologies.

Here, we review the literature on functional characterization of *NAC* genes through over-expression to establish their roles in stress tolerance and biomass production, the two important parameters for biofuel plants.

## NAC Engineered Plants for Growth in Degraded Lands

### Stress Tolerance

Members of *NAC* family genes from different plants have been shown to provide tolerance to biotic and abiotic stresses (Jiang et al., 2014; Mao et al., 2014; Shah et al., 2014). ATAF sub-family in particular is involved in response to environmental stimulus, and the mechanism of action of NAC may be ABA dependent or independent. Ohnishi et al. (2005) provided comprehensive evidence that *OsNAC6* gene in rice was induced by cold, salt, drought, abscisic acid (ABA), jasmonic acid (JA), and wounding. Subsequently, similar results were also reported by Hu et al. (2006, 2008) for *SNAC1* and *SNAC2* genes in rice, which too were induced by cold, drought and salinity stresses. Similarly, *GmNAC11* and *GmNAC20* genes from soybean were shown to be differentially expressed in response to multiple abiotic stress and plant hormones as a transcriptional activator of other stress responsive genes like *DREB1A*, *ERD11*, *cor15A*, *ERF5*, *RAB18*, and *KAT2* genes (Hao et al., 2011). A well-characterized *NAC* gene is *AtNAC2* from *Arabidopsis thaliana*, whose elevated expression levels have been reported in ethylene and auxin overproducer mutants, when exposed to salt stress. The gene also gets induced by ABA and drought stresses (He et al., 2005). In our laboratory, we have identified a cold-inducible *NAC* gene from a Brassicaceae family member *Lepidium latifolium* (*LlaNAC*), which was subsequently validated by over-expressing the gene in tobacco plants (Grover et al., 2014). The popularity of the *NAC* gene as a tool to induce stress tolerance in plants by genetic engineering can well be assessed by overlooking the number of such reports within the calendar year 2015 (**Table 1**).

**Table 1:** Recent reports on demonstration of acquiring stress tolerance in *NAC* over-expressor plants.

Donor plant	Gene	Over-expressor plant	Abiotic stress to which tolerance acquired	Reference
*Macrotyloma uniflorum*	*MuNAC4*	*Arachis hypogaea*	Drought	Pandurangaiah et al., 2014
*Cicer arietinum*	*CarNAC3, CarNAC6*	*Populus deltoides* × *Populus euramericana*	Drought, salinity	Movahedi et al., 2015
*Miscanthus lutarioriparius*	*MlNAC5*	*Arabidopsis thaliana*	Drought, cold	Yang et al., 2015
*Brassica napus*	*BnaNAC19, BnaNAC82*	*Nicotiana benthamiana*	Hypersensitivity-like response to reactive oxygen species	Wang et al., 2015
*Oryza sativa*	*SNAC3*	*Oryza sativa*	Heat, drought	Fang et al., 2015
*Triticum aestivum*	*TaNAC29*	*Arabidopsis thaliana*	Drought, salinity	Huang et al., 2015

From human perspective, a good biofuel crop would be the one which would also be resistant to the abiotic stresses. Interestingly, a small number of NAC proteins have also been reported to respond to the abiotic stresses (reviewed by Nuruzzaman et al., 2013). Examples include *OsNAC111* in rice, belonging to the TERN subgroup, and has been shown to provide tolerance to over-expressor plants against *Magnaporthe oryzae* (Yokotani et al., 2014).

### Physiological and Morphological Adaptations

*NAC* family genes are also involved in a number of growth and development processes, as well as in tissue formation, which in turn help a plant to survive stress. For example, membrane-associated NAC TFs, up-regulated by stress conditions, have been found associated to a variety of morphological features like delayed flowering, reduced growth, curled leaves, etc., under stress conditions (Kim et al., 2007a,b). *Arabidopsis* lines over-expressing *RhNAC3* gene from rose displayed hypersensitivity during seed germination and leaf closure on ABA or drought stresses (Jiang et al., 2014). A number of *NAC* genes have also been reported downstream to ETHYLENE-INSENSITIVE2 or similar genes, thereby participating in leaf senescence, fruit ripening, etc. (Ay et al., 2014; Kim et al., 2014; Nieuwenhuizen et al., 2015). Zhao et al. (2015) reported delayed leaf senescence and higher nitrogen concentrations in grain by over-expressing wheat *TaNAC-S.*

Another well known effect of *NAC* over-expression in plants is lateral root formation, which is generally observed as a response or phenotypic adaptation to water scarcity. The effect has best been studied for *AtNAC*2 from *Arabidopsis thaliana*, being associated to lateral root development under salt stress. Further, model plants over-expressing *AtNAC2* gene were also shown to have extensive lateral root development (He et al., 2005).

## NAC Engineered Plants for Biomass Production

Biomass, i.e., deposition of photosynthetic free energy, is an important source of biofuels today. Vegetative tissues of specialized crops like switch grass, miscanthus and poplar are primarily used for this purpose. Therefore, any engineering event in plants that prolongs or enhances vegetative meristematic activity is desirable from biomass production point of view. In our laboratory, we raised *LlaNAC* over-expressor lines that could accumulate 2–3 times more biomass and chlorophyll pigments than the wild-types. In addition, these plants matured early, had shorter life cycles (Grover et al., 2014), and could capture 3–5 times more carbon dioxide. It is pertinent to mention that the nearest homolog, ANAC056 from *Arabidopsis thaliana*, of the gene that we cloned (i.e., *LlaNAC*) clustered with VND subfamily of genes (Zhong et al., 2010a). Expression of ANAC056 is predominant in cork, xylem, silique, hypocotyls, and stamen. VND genes clearly participate in secondary growth in perennials, their overall effects in annuals and herbaceous plants like *Arabidopsis* and *Lepidium*, shall be more thoroughly evaluated. VND proteins function in formation of various tissues other than xylem vessels too (Bennett et al., 2010).

NAC proteins target a number of genes in the genome including those which are involved in stress responses, growth, and secondary wall synthesis (discussed below) or cambial activities. Previous estimated have suggested that as many as 72 genes are the target genes to NAC proteins (Shamimuzzaman and Vodkin, 2013). Such versatility in their action is partly due to their ability to form homo- and hetero-dimers, which act as transcriptional switches (Olsen et al., 2005), and partly because many *NAC* TFs are downstream to each other (Grover et al., 2014).

### Secondary Cell Wall Synthesis

Secondary cell walls are the most abundant biomass and renewable source of energy. Interestingly, secondary cell wall biosynthesis is regulated by a subset of closely related NAC domain proteins, i.e., NST1/ANAC043, NST2/ANAC066, and NST3/SND1 (SECONDARY WALL-ASSOCIATED NAC DOMAIN PROTEIN1)/ANAC012 as master transcriptional switches (Zhong et al., 2008). These proteins bind to a triggering expression of other genes downstream by binding at 19-bp consensus sequence, (T/A)NN(C/T)(T/C/G)TNNNNNNNA(A/C)GN(A/C/T)(A/T) called as secondary wall NAC binding element (SNBE; Zhong et al., 2010b). Downstream genes, *SND2* and *SND3* (Zhong et al., 2008) up-regulate genes associated with cellulose, xylan, mannan, and lignin biosynthesis and polymerization.

Other NAC TFs belonging to this sub-family include Vascular NAC related domain proteins (VND6 and VND7) which act as regulators of secondary cell wall biosynthesis specifically in vascular vessels (Yamaguchi et al., 2008). These genes are expressed in fiber cells of inflorescence stems, hypocotyls, valve endocarp layer and in the cells surrounding vascular vessels in replum of siliques (Shah et al., 2014). Examples include BdSWN5 in *Brachypodium distachyon*, OsSWN1 in grasses (Chai et al., 2015), and wood associated NAC domain proteins (WND2B and WND6B) in poplar (Zhong et al., 2010a; Zhao et al., 2014). The role of alternative splicing too has been suggested in function of these genes as regulators of secondary cell wall biosynthesis (Zhao et al., 2014). Interestingly, a number of other *NAC* genes too lie downstream of *VND* subfamily genes (Shamimuzzaman and Vodkin, 2013). Wood-associated NAC domain TF (PtrWNDs), are alternatively spliced (Zhao et al., 2014) occurs exclusively in secondary xylem fiber cells. The two PtrWND1B isoforms play antagonistic roles in regulating cell wall thickening during fiber cell differentiation in *Populus* sp.

Cellular maturation of root cap is also regulated by NAC TF family members, viz., *SMB* (*SOMBRERO*), *BRN1* (*BEARSKIN1*), and *BRN2*, along with *VND* and *NST* genes (Bennett et al., 2010). Interestingly, *SMB*, *BRN1*, and *BRN2* over-expression show similar phenotypic patterns to *VND/NST* genes (Bennett et al., 2010).

## Perspectives

*NAC* gene family is not only one of the largest gene families in plants, but is also one of the best characterized gene families in plants. A random search on google scholar using the key words “Characterization of *NAC* genes” returns more than 100 relevant result links. Inclusion of information from all of these papers would have required publication of a monograph on *NAC* genes, and is thus out of the scope of this mini-review. However, there have been few or even negligible efforts on realization of potential of the *NAC* genes by preparing genetically engineered crops for food or fuel purposes. Thus, while *NAC* genes remain a favorite among Plant Molecular Biologists, they are yet to be adopted by Plant Biotechnologists.

Sufficient evidence is available on, how *NAC* over-expressing plants negotiate stress better than the wild-type plants. A transgenic crop over-expressing *NAC* gene would thus have much wider applicability and adaptability to a conventional breed or variety. Conversely, the agrochemicals can judiciously be directed to food crops. Further, a number of reports implicating role of NAC proteins in secondary wall synthesis and thereby biomass accumulation are available, thereby opening a lucrative opportunity for designing second or fourth generation biofuel plants. An obvious objection would be that stress tolerance and biomass accumulation are different properties, and are not accounted by a single *NAC* gene. Panorama of traits are generally observed in transgenic plants designed with an objective to characterize these genes. For example, Xia et al. (2010) observed biotic and abiotic stress tolerance due to the effect of a single gene, Uauy et al. (2006) reported senescence controlling *NAC* gene improving grain protein, zinc, and iron content in wheat, *NAC* gene from *Lepidium latifolium* improved both stress tolerance as well as biomass characters in over-expressing tobacco lines (Grover et al., 2014). Much recently, Chen et al. (2015) found *OsNAC2* over-expression affects height of the plant, insensitivity to gibberellic acid and delays flowering.

In view of the above, it is justified to conclude that an open minded screening of NAC over-expressor plants is required to evaluate the potential for biofuel plants. Implementation of NAC transgenic plants for economic benefits in biofuel production may be dictated by the local requirements, and necessary strategy can be drafted based on the huge literature available in public domain.

## Author Contributions

SS conceived the idea. SS and AG built the article together, and MN approved the final version.

## Conflict of Interest Statement

The authors declare that the research was conducted in the absence of any commercial or financial relationships that could be construed as a potential conflict of interest.
